# Treatment of Hepatitis C Virus Infections Among Patients of Ukrainian Origin During the Influx of War Refugees to Poland

**DOI:** 10.3390/jcm13247641

**Published:** 2024-12-15

**Authors:** Robert Flisiak, Dorota Zarębska-Michaluk, Diana Martonik, Justyna Janocha-Litwin, Hanna Berak, Marek Sitko, Włodzimierz Mazur, Ewa Janczewska, Beata Lorenc, Jakub Klapaczyński, Łukasz Laurans, Dorota Dybowska, Anna Piekarska, Magdalena Tudrujek-Zdunek, Krystyna Dobrowolska, Anna Parfieniuk-Kowerda

**Affiliations:** 1 Department of Infectious Diseases and Hepatology, Medical University of Białystok, 15-540 Białystok, Poland; robert.flisiak1@gmail.com (R.F.); diana.martonik@umb.edu.pl (D.M.); anna.parfieniuk@gmail.com (A.P.-K.); 2 Department of Infectious Diseases and Allergology, Jan Kochanowski University, 25-317 Kielce, Poland; dorota1010@tlen.pl; 3 Department of Infectious Diseases and Hepatology, Wrocław Medical University, 50-367 Wrocław, Poland; justyna@janocha-litwin.pl; 4 Outpatient Clinic, Hospital for Infectious Diseases in Warsaw, 01-201 Warsaw, Poland; hberak@zakazny.pl; 5 Department of Infectious and Tropical Diseases, Jagiellonian University, 31-008 Kraków, Poland; sitkomar@o2.pl; 6 Clinical Department of Infectious Diseases, Medical University of Silesia, 41-500 Chorzów, Poland; wlodek.maz@gmail.com; 7 Department of Basic Medical Sciences, School of Public Health in Bytom, Medical University of Silesia, 40-055 Katowice, Poland; e.janczewska@poczta.fm; 8 Pomeranian Center of Infectious Diseases, Medical University Gdańsk, 80-214 Gdańsk, Poland; lormar@gumed.edu.pl; 9 Department of Internal Medicine and Hepatology, National Medical Institute of the Ministry of the Interior and Administration, 02-507 Warsaw, Poland; klapaj@gmail.com; 10 Department of Infectious Diseases, Hepatology and Liver Transplantation, Pomeranian Medical University, 70-204 Szczecin, Poland; asklepiada@wp.pl; 11 Department of Infectious Diseases and Hepatology, Faculty of Medicine, Nicolaus Copernicus University, 85-030 Bydgoszcz, Poland; d.dybowska@wsoz.pl; 12 Department of Infectious Diseases and Hepatology, Medical University of Łódź, 90–419 Łódź, Poland; annapiekar@gmail.com; 13 Department of Infectious Diseases, Medical University of Lublin, 20-059 Lublin, Poland; magdalena.tudrujek@gmail.com; 14 Collegium Medicum, Jan Kochanowski University, 25-317 Kielce, Poland

**Keywords:** migration, HCV, hepatitis c, direct-acting antivirals

## Abstract

**Background:** The wave of wartime migration from Ukraine has raised a number of concerns about infectious diseases, the prevalence of which is higher in Ukraine than in host countries, with hepatitis C virus (HCV) infection being one of them. Our analysis aimed to assess the percentage of HCV-infected Ukrainian refugees under care in Polish centers providing antiviral diagnosis and therapy, to evaluate their characteristics and the effectiveness of treatment with direct-acting antiviral drugs (DAAs). **Methods:** The analysis included patients of Polish and Ukrainian nationality treated for HCV infection between 2022 and 2024 in Polish hepatology centers. Data were collected retrospectively and completed online. **Results:** In the population of 3911 patients with chronic hepatitis C treated with DAAs in 16 Polish centers in 2022–2024, there were 429 war refugees from Ukraine, accounting for 11% of the total treated. The Ukrainian population was significantly younger (45.7 vs. 51 years, *p* < 0.001) and had a higher percentage of women (50.3% vs. 45.3%, *p* = 0.048) compared to Polish patients. Patients of Ukrainian origin had less advanced liver disease and were significantly less likely to have comorbidities and the need for comedications. Coinfection with human immunodeficiency virus was significantly more common in Ukrainians than in Polish patients, 16.1% vs. 5.9% (*p* < 0.001). The distribution of HCV genotypes (GTs) also differed; although GT1b predominated in both populations, its frequency was significantly higher in the Polish population (62.3% vs. 44.5%, *p* < 0.001), while the second most common GT3 was significantly more common in Ukrainian patients (30.5% vs. 16.2%, *p* < 0.001). **Conclusions**: Documented differences in patient characteristics did not affect the effectiveness of antiviral therapy, which exceeded 97% in both populations, but there was a higher rate of those lost to follow-up among Ukrainian patients.

## 1. Introduction

Russian aggression against Ukraine has caused the largest migration crisis in Europe since World War II. Millions of Ukrainian citizens have been forced to leave their homeland in search of safety in other European countries. Among the countries where the largest number of war refugees have arrived was Poland. According to estimates by the border guards, several million Ukrainians have entered the country since the beginning of the war. For some of them, it was a transit point on their onward journey; some decided to return to their homeland; and for about a million, Poland became the final destination. Importantly, along with the people came their health problems and diseases they suffer from, of which infectious illnesses have a significant share. Differences in the prevalence of certain communicable diseases in Ukraine and Poland have influenced their epidemiology in our country, especially with regard to infection with human immunodeficiency virus (HIV), tuberculosis, sexually transmitted diseases, and hepatitis C [1,2].

Based on available data, Ukraine has one of the highest prevalences of HCV infection in the world, with 3.1% of viremic cases, corresponding to about 1,300,000 infected patients [3,4]. In high-risk populations, it is even much higher exceeding 60% among drug users [4,5]. However, according to the Ministry of Health of Ukraine and patient organizations, the actual incidence is much higher than the official rate, reaching 5%, and only 7% of infected people are aware of the disease and receive the necessary specialized healthcare [6,7]. In November 2019, Ukraine joined the global strategy of the World Health Organization (WHO) to eliminate hepatotropic virus infections as a public health threat by adopting a national strategy to combat viral hepatitis [8]. As a result of taking up this challenge, 230 healthcare institutions have contracted state-funded antiviral treatment free of charge to patients [9]. These facilities provided about 16,000 thousand therapies annually, which did not meet the need [9]. The situation was significantly worsened by the Russian aggression against Ukraine, during which, due to war damage and the reorganization of the state budget, there were only 80 facilities left that could provide HCV diagnostics and treatment to about 10,000 patients a year [9]. Reaching medical institutions has become an additional difficulty at a time when survival has become more important than health.

This priority was the reason for a huge wave of refugees, which also included patients infected with HCV, both aware of it and undiagnosed. Addressing this issue, a joint statement from the European Center for Disease Prevention and Control (ECDC), WHO, and the European Association for the Study of the Liver (EASL) has been issued, reviewing public health guidelines aimed at ensuring that the needs of refugees with viral hepatitis are adequately met across the continuum of care, from prevention to treatment in host countries [10]. In Poland, thanks to a special law on assistance to Ukrainian citizens in connection with the armed conflict that grants the right to health services to war refugees from Ukraine on the same terms as insured citizens of Poland, they have gained access to diagnosis and treatment of acute and chronic diseases, including HCV infection [11].

The aim of our analysis was to assess the percentage of Ukrainians infected with HCV treated in Polish centers providing antiviral diagnostics and therapy to evaluate their characteristics and the effectiveness of treatment with direct-acting antiviral drugs (DAAs) compared to Polish patients.

## 2. Materials and Methods

This study included patients from the EpiTer-2 database, which is an ongoing, retrospective, multicenter, national study initiated by investigators from the Polish Association of Epidemiologists and Infectious Disease Physicians, conducted in real-world experience conditions, assessing antiviral treatment in 19,441 patients with chronic hepatitis C treated in 22 Polish hepatology centers between 2015 and 2024. The current analysis included 3911 patients from 16 centers that were able to determine the Polish or Ukrainian nationality of patients treated for HCV infection between 2022 and 2024.

The type of antiviral treatment used was selected by the attending physician based on current national recommendations [12] and the reimbursement policy of the National Health Fund (NFZ). Doses and duration of treatment were prescribed in accordance with the Summary of Product Characteristics of the individual drugs.

All patients gave informed consent before starting treatment according to the requirements of the National Health Fund. Clinical and laboratory data were collected retrospectively and sent via an online platform operated by Tiba sp. z o.o. (Wrocław, Poland) in line with the national regulations on protecting personal data in Poland. The originally collected data were used to monitor the effectiveness and safety of registered drugs in real clinical conditions under the requirements of the National Health Fund and were not intended for scientific purposes. Patients were not subjected to any experimental procedures or therapies, which meant that the EpiTer-2 program was non-interventional and, according to the law in effect at the time of its implementation, did not require ethics committee approval.

Baseline data included age, sex, body mass index (BMI), comorbidities and concomitant medications, assessment of liver disease severity, hepatitis B virus (HBV) and human immunodeficiency virus (HIV) coinfection, and history of previous antiviral therapy. Liver disease severity was assessed by transient elastography (TE) performed with a FibroScan device (Echosens, Paris, France), shear wave elastography (SWE) using the Aixplorer system (SuperSonic Imagine, Aix-en-Provence, France), or liver biopsy. Results were standardized to the METAVIR score (F0–F4) using the EASL recommendations. Patients with cirrhosis were assessed using the Child–Pugh (CP) and Model for End-of-Life Liver Disease (MELD) scores. HCV RNA was assessed by real-time polymerase chain reaction assays with a lower limit of detection at 15 IU/mL. Effectiveness assessment was performed 12 weeks after the end of treatment using an intent-to-treat (ITT) analysis, including patients who received at least one dose of antiviral therapy, and a per-protocol (PP) analysis, including only patients who were not lost to follow-up.

The results were expressed as mean with standard deviation or absolute number objectified by percentage values. Statistical analysis was performed with GraphPad (Prism Software, Prism 10, Irvine, CA, USA). The data were assessed for normality using the Shapiro–Wilk test, and then comparative analysis between groups was carried out by Mann–Whitney U test for continuous data and chi-square for categorical data. The differences were considered statistically significant if *p* < 0.05.

## 3. Results

Of the 3911 individuals included in this study, 429 (11%) were patients of Ukrainian origin. As can be seen from Table 1, the highest percentage of patients of Ukrainian origin among all treated patients, even above 20%, was recorded in centers located in northern (Szczecin, Gdańsk) and southern Poland (Kraków, Wrocław, Chorzów, Myślenice), while the lowest, below 3%, was in the eastern part of the country (Białystok, Lublin).

In contrast to Polish patients, the Ukrainian population was significantly younger (45.7 years vs. 51 years, *p* < 0.001) and had a higher proportion of females (50.3% vs. 45.3%, *p* = 0.048). No significant differences were observed in BMI, and the percentages of patients who did not respond to previous treatment were similar (Table 2).

**Figure 1:**
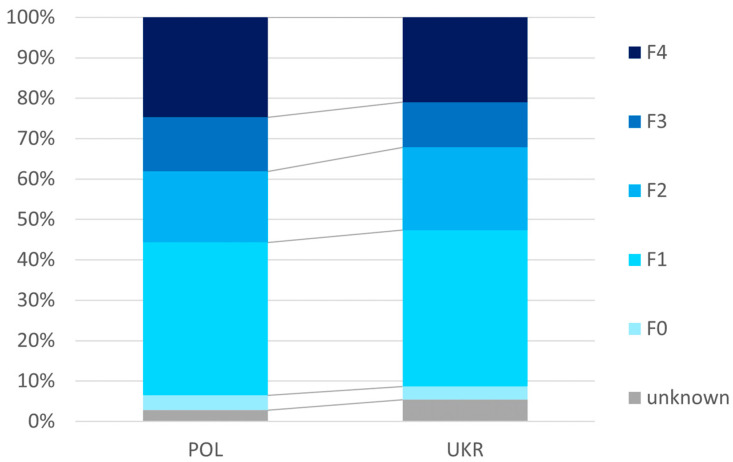
Advancement of liver disease.

As shown in Figure 1, the Ukrainian population was characterized by less advanced liver disease, as expressed by a significantly (*p* = 0.017) lower percentage of cumulative patients with F3 or F4 (32.2% vs. 38.1%), as well as significantly lower mean values of liver tissue stiffness assessed by elastography (10.3 ± 9.7 vs. 12.0 ± 11.4 kPa) and MELD score (7.6 ± 2.5 vs. 7.8 ± 2.5) (Table 3).

As can be seen from Table 2 and Table 3, patients of Ukrainian origin had significantly less frequent comorbidities and required additional medications and less frequently had a history of hepatocellular carcinoma and decompensation in the past, as well as the presence of esophageal varices or ascites at the time of starting therapy. HIV coinfection was reported significantly more often in the Ukrainian population, while the incidence of HBV coinfection was comparable (Table 2).

**Figure 2:**
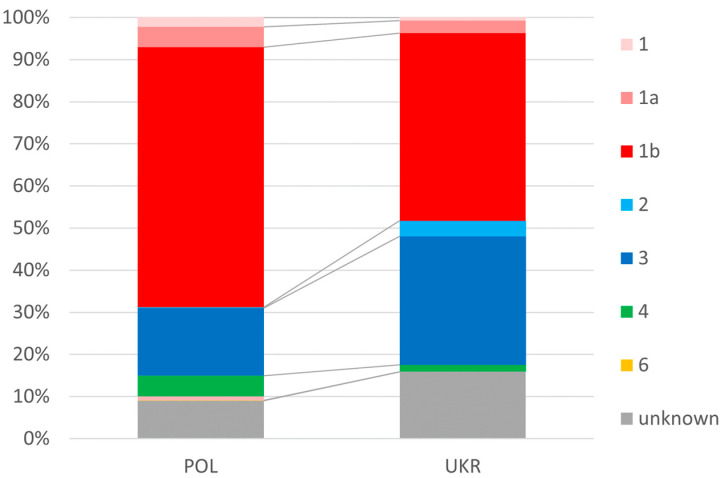
Prevalence of genotypes.

In the Ukrainian population of patients, infections with genotypes 2 and 3 occurred significantly more frequently than in the Polish population (34.2% vs. 21.1%), and genotype 1 was less frequent (48.2% vs. 69.4%) (Figure 2).

As can be seen from Table 3, the values of laboratory parameters analyzed before the start of treatment demonstrated some statistically significant differences, including higher ALT activity (97.1 vs. 88.7 IU/L, *p* = 0.02), higher albumin concentration (4.3 vs. 4.2 g/dL, *p* = 0.001), and lower bilirubin concentration (0.74 vs. 0.81 mg/dL, *p* = 0.01) in Ukrainians as compared to Polish patients.

Regardless of nationality, patients received similar types of therapy, with glecaprevir/pibrentasvir being the most common (Table 4).

**Figure 3:**
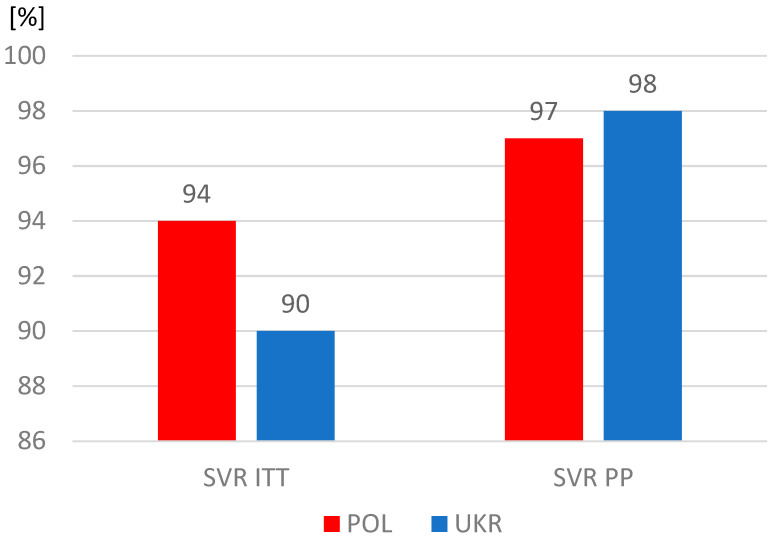
Sustained virologic response (SVR); ITT, intent-to-treat analysis; PP, per protocol analysis.

The proportions of patients who completed therapy according to plan were similar, but significantly more often patients from Ukraine did not report for a visit assessing the effectiveness of therapy and were considered lost to follow-up. As a result, the sustained virologic response (SVR) rate among Ukrainians was significantly lower in the intent-to-treat analysis, which included all patients who started treatment, including those lost to follow-up (Table 4). However, after excluding this group, the SVR values calculated per protocol exceeded 97% and were similar in both study populations (Figure 3).

## 4. Discussion

The wave of war refugees from Ukraine flowing widely through many European countries has raised a number of concerns about the transmission of infectious diseases, the incidence of which has been higher in Ukraine than in host countries [13,14]. The insufficient level of vaccination against many communicable diseases in Ukraine and the sanitary conditions in which refugees traveled and stayed led to the list of concerns starting with acute vaccine-preventable diseases, such as measles, rubella, whooping cough, diphtheria, polio, hepatitis B (HBV) and tuberculosis [15,16,17]. However, the wave of migration also carried the risk of infections that cannot be prevented by vaccines and whose incidence in Ukraine was significantly higher than in the host countries, including sexually transmitted diseases, HIV, and HCV infection [18,19]. Awareness of this risk was reflected in the joint position of WHO, ECDC, and EASL, whose goal was to provide the necessary diagnostic and therapeutic care to refugees with hepatotropic virus infections [10]. Solutions regulating medical care in host countries were designed in different ways; in Poland, people from Ukraine were provided with free medical care on the basis of the so-called special law that made diagnosis and therapy possible for such patients [13].

Testing of Ukrainian refugees in Poland for the presence of anti-HCV antibodies is carried out on a voluntary basis. HCV infection diagnostic and treatment centers receive both newly diagnosed patients and those who were previously aware of the infection, but due to limited access to DAA therapy in Ukraine, have not yet started treatment. In practice, medical care focuses on qualification for therapy and antiviral treatment, while cases of continuation of DAA administration started in Ukraine seem to be marginal due to the short duration of treatment.

The subjects of our analysis, which covered 15 Polish cities, were refugees from Ukraine with chronic hepatitis C undergoing antiviral therapy with DAAs. They accounted for a total of 11% of all those receiving antiviral treatment for HCV infection in these centers, noting that the spread of their share from center to center was very large, ranging from less than 3 to more than 21%. The highest percentage of Ukrainian patients was reported in large hepatology centers in the north and south of Poland, in cities with high potential for residence and employment. Not insignificant for some of these centers seems to be the location near Poland’s western border with Germany, which is a further destination for many refugees from Ukraine [20]. Although the largest Ukrainian population resides in Warsaw and the Mazovia province, the percentage of Ukrainians treated with DAAs in these areas was just over 11%, perhaps because our analysis did not include all the centers located in this area. The lowest percentage of Ukrainian patients were treated in hepatology centers in eastern Poland, which seems to be the least attractive for refugees in terms of residence and profitable work.

Nevertheless, based on these data, the increase in demand for DAA therapy in Polish centers caused by the wave of migration from Ukraine did not reach the scale as predicted, taking into account the several-fold difference in the prevalence of HCV. One of the possible reasons can be refugees’ fears of discrimination against infected people, social stigmatization in a new living environment, and the risk of losing their jobs; the language barrier also matters [21]. Fear of discrimination in the community and workplace was particularly pronounced for individuals with HIV. Although a significantly higher rate of HIV/HCV coinfection was documented in our study among Ukrainian refugees compared to Polish patients treated with DAAs, the difference was not as high as one would expect based on available data [19]. The main route of HCV transmission in Ukraine is intravenous drug use, and among the estimated 340,000 PWID (people who inject drugs) in Ukraine, the prevalence of HCV exceeds 50%, which is even higher in selected cities such as Kyiv with 84.8%; more than half of the cases involve coinfection with HIV, while in our analysis, they accounted for only 16% compared to 6% in the Polish patient group [19,22]. However, the incidence of HBV coinfection was comparable in both nations, amounting to less than 1%, and in the case of Polish patients, it was consistent with previously published data [23]. In the case of Ukraine, only data on the population prevalence of HBsAg obtained by modeling are available, reporting a value of 1% [24].

When comparing the HCV-infected populations from both countries, we noticed differences in the age of treated patients; individuals from Ukraine were significantly younger than those from Poland. It is difficult to comment on these data, as there is a lack of analyses describing HCV-infected patients in Ukraine; data from a single publication on a population of 868 patients, mostly PWID (87%), 55% of whom were coinfected with HIV, report a mean age of 39 years at the start of DAA treatment [25]. The latest published Polish data indicate that the largest percentage of patients diagnosed with HCV infection, which in practice equates to almost simultaneous qualification for therapy and immediate initiation of treatment, is in the 45–64 age range [26].

The lower age of Ukrainian patients should probably be linked to the lower burden of comorbidities and the medications taken for them as compared to the Polish population. The lower age of patients from Ukraine also explains why they were characterized by less advanced liver disease, defined by the percentage of individuals with advanced liver fibrosis and cirrhosis and a lower share of those with decompensation of liver function, both in history and at the start of therapy. A smaller percentage of refugees had a history of hepatocellular carcinoma (HCC) and documented esophageal varices. Less advanced liver disease in the refugee population also explains the significant differences in laboratory parameters, such as albumin and bilirubin levels.

The phenomenon of less severe liver disease may have been influenced not only by the lower age of Ukrainian patients but also by difficulties in reaching healthcare facilities for older refugees, for whom the language and information barrier is greater than in the case of younger people [21].

Another significant difference that we reported in our analysis concerned the gender distribution of infected patients. The Polish population was dominated by men, constituting 55%, and among patients from Ukraine, women predominated, with a 50.3% share. While we expected a predominance of women in the Ukrainian population, its small scale was a surprise to us, considering that approximately 80% of war refugees from Ukraine are women [27]. One of the possible explanations for this disproportion is that the domination of women among refugees was offset by the significant predominance of men with chronic hepatitis C; according to available data, they constitute more than 60% of all HCV-infected people in Ukraine [25,28].

In contrast, we were not surprised by the differences in the distribution of HCV genotypes documented in our study. In both populations, genotype 1 (GT1) dominated, but the dominance was more pronounced in the Polish population, with almost 70% compared to 48% in Ukrainians. This order and the smaller difference between GT1 and the second most common GT3 in the Ukrainian population compared to Polish patients are consistent with published data [28,29,30].

The differences identified in our analysis in the characteristics of the populations of Polish and Ukrainian patients with HCV infection did not translate into treatment effectiveness, which exceeded 97% in both groups. It should be noted, however, that with a comparable percentage of people completing therapy as planned, the percentage of patients lost to follow-up among Ukrainian refugees was twice as high as that in the Polish group. It seems that this may have been partly due to an unstable life situation, forcing a return to their homeland or further migration. On the other hand, the fact that as many as 92% of patients remained under observation, usually lasting 20 to 24 weeks, means that when starting treatment, they planned to stay longer or permanently in Poland.

Our study has limitations that we have to declare. Some of these are due to the real-world nature and retrospective design of the analysis and involve possible bias, incomplete data, and errors at the database entry stage. The study did not cover all centers treating HCV infection in Poland and thus certainly did not include all war refugees from Ukraine treated in our country. However, the analyzed group was diverse and encompassed different regions of Poland, thus becoming a representative sample, which we consider its strength. To the best of our knowledge, this is the first analysis of DAA therapy in HCV-infected war refugees from Ukraine in the host country. Although our analysis focuses on migrants from Ukraine in Poland, we believe that the findings we obtained may be useful for the elimination of HCV infection in this population in other host countries as well, as the wave of migration from Ukraine sweeps through many European countries.

## 5. Conclusions

An analysis of nearly 4000 HCV-infected patients treated with DAAs in several centers in various regions of Poland revealed an 11% share of refugees from Ukraine. Patients of Ukrainian origin were younger and had fewer comorbidities and less advanced liver disease compared to Polish patients, but they were significantly more often coinfected with HIV. The described differences in patient characteristics did not affect the effectiveness of antiviral therapy, which exceeded 97% regardless of the origin of the patients, but among Ukrainian patients, there was a higher percentage of people lost to follow-up without SVR assessment.

## Figures and Tables

**Table 1:** Distribution of Ukrainian patients in the treating centers.

	UKR	POL	ALL	% UKR
Szczecin	21	76	97	21.6%
Kraków	58	279	337	17.2%
Gdańsk	36	193	229	15.7%
Warszawa NMIMIA	22	124	146	15.1%
Gorzów	10	57	67	14.9%
Wrocław	72	441	513	14.0%
Chorzów	43	270	313	13.7%
Mysłowice	42	282	324	13.0%
Łódź	11	78	89	12.4%
Warszawa HID	71	589	660	10.8%
Olsztyn	6	82	88	6.8%
Bydgoszcz	12	169	181	6.6%
Bytom	3	83	86	3.5%
Kielce	7	203	210	3.3%
Lublin	10	351	361	2.8%
Białystok	5	205	210	2.4%
SUM	429	3482	3911	11.0%

Abbreviations: UKR, Ukraine; POL, Poland; NMIMIA, National Medical Institute of the Ministry of the Interior and Administration; HID, Hospital for Infectious Diseases.

**Table 2:** Patients characteristics.

	POL, N = 3482	UKR, N = 429	*p*
Females/Males, n (%)	1578/1904 (45.3/54.7)	216/213 (50.3/49.7)	0.048
Age, years, mean ± SD	51.0 ± 14.0	45.7 ± 10.6	<0.001
BMI, mean ± SD	26.1 ± 4.5	25.8 ± 4.2	0.274
BMI, n (%)			
<18.50	73 (2.1)	11 (2.6)	0.529
18.50–24.99	1443 (41.4)	187 (43.6)	0.395
25.00–29.99	1366 (39.2)	163 (38.0)	0.621
>29.99	590 (16.9)	66 (15.4)	0.415
Unknown	10 (0.3)	2 (0.5)	0.527
Failure of previous HCV therapy, n (%)	155 (4.5)	18 (4.2)	0.788
History of hepatocellular carcinoma, n (%)	61 (1.8)	3 (0.7)	0.107
History of hepatic decompensation, n (%)	112 (3.2)	9 (2.1)	0.208
History of liver transplantation, n (%)	6 (0.2)	1 (0.2)	0.782
History of kidney transplantation, n (%)	7 (0.2)	1 (0.2)	0.891
HIV coinfection, n (%)	204 (5.9)	69 (16.1)	<0.001
HBV coinfection (HBsAg+), n (%)	27 (0.8)	4 (0.9)	0.667
Comorbidities, n (%)	2192 (63.0)	172 (40.1)	<0.001
Concomitant medication, n (%)	2077 (59.6)	188 (43.8)	<0.001

Abbreviations: BMI, body mass index; HBV, hepatitis B virus; HCV, hepatitis C virus; HIV, human immunodeficiency virus; SD, standard deviation; HBsAg, hepatitis B virus surface antigen.

**Table 3:** Characteristics of the HCV infection and liver disease.

	POL, N = 3482	UKR, N = 429	*p*
Genotypes, n (%)			
1	75 (2.2)	3 (0.7)	<0.001
1a	172 (4.9)	13 (3.0)	0.079
1b	2170 (62.3)	191 (44.5)	<0.001
2	8 (0.2)	16 (3.7)	<0.001
3	563 (16.2)	131 (30.5)	<0.001
4	172 (4.9)	7 (1.6)	0.002
6	4 (0.1)	0	0.482
Unknown	318 (9.1)	68 (15.9)	<0.001
Laboratory measures, mean ± SD			
HCV RNA, IU/mL	2.9 ± 9.3 × 10^6^	2.7 ± 4.4 × 10^6^	0.247
ALT, IU/L	88.7 ± 80.7	97.1 ± 83.1	0.002
Albumins, g/dL	4.2 ± 0.5	4.3 ± 0.5	0.001
Bilirubin, mg/dL	0.81 ± 0.74	0.74 ± 0.56	0.010
Platelets, 1/μL	206,319 ± 79,332	207,263 ± 81,360	0.667
Creatinine, mg/dL	0.84 ± 0.43	0.89 ± 0.92	0.121
Disease advancement, n (%)			
F0	128 (3.7)	14 (3.3)	0.667
F1	1317 (37.8)	166 (38.7)	0.726
F2	614 (17.6)	88 (20.5)	0.143
F3	467 (13.4)	48 (11.2)	0.199 *
F4	859 (24.7)	90 (21.0)	0.092 *
Unknown	97 (2.8)	23 (5.4)	0.004
Liver stiffness, kPa, mean ± SD	12.0 ± 11.4	10.3 ± 9.7	0.008
Esophageal varices, n (%)	182 (5.2)	12 (2.8)	0.045
Ascites at the start of treatment, n (%)	83 (2.4)	4 (0.9)	0.054
MELD, mean ± SD	7.8 ± 2.5	7.6 ± 2.5	0.012
MELD > 15, n (%)	122 (3.5)	12 (3.4)	0.447
Child–Pugh B or C, n (%)	148 (4.2)	12 (2.8)	0.150

Abbreviations: ALT, alanine aminotransferase; HCV, hepatitis c virus; SD, standard deviation; RNA, ribonucleic acid; * statistical significance (*p* = 0.017) achieved for cumulative data of F3 and F4.

**Table 4:** Therapy and its effectiveness.

	POL, N = 3482	UKR, N = 429	*p*
Regimens, n (%)			
GLE/PIB	1845 (53.0)	238 (55.5)	0.332
SOF based	1588 (45.6)	187 (43.6)	0.426
Other	47 (1.4)	4 (0.9)	0.446
Course of the treatment, n (%)			
Completed	3401 (97.7)	417 (97.2)	0.546
Modified	8 (0.2)	0	0.320
Discontinued	37 (1.1)	3 (0.7)	0.480
Unknown	36 (1.0)	9 (2.1)	0.051
Adverse events, n (%)	241 (6.9)	21 (4.9)	0.113
Deaths, n (%)	28 (0.8)	2 (0.5)	0.457
Lost to follow-up, n (%)	135 (3.9)	33 (7.7)	<0.001
SVR ITT, n (%)	3259/3482 (93.6)	387/429 (90.2)	0.001
SVR PP, n (%)	3259/3347 (97.4)	387/396 (97.7)	0.673

Abbreviations: GLE, glecaprevir; PIB, pibrentasvir; SOF, sofosbuvir; SVR, sustained virologic response; ITT, intent to treat; PP, per protocol.

## Data Availability

The data that support the findings of this study are available upon request from the corresponding author. The data are not publicly available due to privacy reasons.
